# T-cell responses targeting HIV Nef uniquely correlate with infected cell frequencies after long-term antiretroviral therapy

**DOI:** 10.1371/journal.ppat.1006629

**Published:** 2017-09-20

**Authors:** Allison S. Thomas, Kimberley L. Jones, Rajesh T. Gandhi, Deborah K. McMahon, Joshua C. Cyktor, Dora Chan, Szu-Han Huang, Ronald Truong, Alberto Bosque, Amanda B. Macedo, Colin Kovacs, Erika Benko, Joseph J. Eron, Ronald J. Bosch, Christina M. Lalama, Samuel Simmens, Bruce D. Walker, John W. Mellors, R. Brad Jones

**Affiliations:** 1 Department of Microbiology Immunology and Tropical Medicine, George Washington University, Washington, District of Columbia, United States of America; 2 Ragon Institute of MIT, MGH, and Harvard, Cambridge MA, United States of America; 3 Massachusetts General Hospital, Boston, Massachusetts, United States of America; 4 Division of Infectious Diseases, Department of Medicine, University of Pittsburgh School of Medicine, Pittsburgh, Pennsylvania, United States of America; 5 Maple Leaf Medical Clinic, Toronto, Canada; 6 Division of Infectious Diseases, Department of Medicine, University of North Carolina School of Medicine, Chapel Hill, North Carolina, United States of America; 7 Harvard TH Chan School of Public Health, Boston, Massachusetts, United States of America; 8 Department of Epidemiology and Biostatistics, George Washington University, Milken Institute School of Public Health, Washington, District of Columbia, United States of America; 9 Howard Hughes Medical Institute, Chevy Chase, Maryland, United States of America; Miller School of Medicine, UNITED STATES

## Abstract

HIV-specific CD8^+^ T-cell responses limit viral replication in untreated infection. After the initiation of antiretroviral therapy (ART), these responses decay and the infected cell population that remains is commonly considered to be invisible to T-cells. We hypothesized that HIV antigen recognition may persist in ART-treated individuals due to low-level or episodic protein expression. We posited that if persistent recognition were occurring it would be preferentially directed against the early HIV gene products Nef, Tat, and Rev as compared to late gene products, such as Gag, Pol, and Env, which have higher barriers to expression. Using a primary cell model of latency, we observed that a Nef-specific CD8^+^ T-cell clone exhibited low-level recognition of infected cells prior to reactivation and robust recognition shortly thereafter. A Gag-specific CD8^+^ T-cell clone failed to recognized infected cells under these conditions, corresponding with a lack of detectable Gag expression. We measured HIV-specific T-cell responses in 96 individuals who had been suppressed on ART for a median of 7 years, and observed a significant, direct correlation between cell-associated HIV DNA levels and magnitudes of IFN-γ-producing Nef/Tat/Rev-specific T-cell responses. This correlation was confirmed in an independent cohort (n = 18). Correlations were not detected between measures of HIV persistence and T-cell responses to other HIV antigens. The correlation with Nef/Tat/Rev-specific T-cells was attributable to Nef-specific responses, the breadth of which also correlated with HIV DNA levels. These results suggest that ongoing Nef expression in ART-treated individuals drives preferential maintenance and/or expansion of T-cells reactive to this protein, implying sensing of infected cells by the immune system. The direct correlation, however, suggests that recognition does not result in efficient elimination of infected cells. These results raise the possibility that enhancing the cytolytic activity of Nef-specific T-cells may lead to reductions in infected cell frequencies, even in the absence of therapeutic latency reversal.

## Author summary

Antiretroviral therapy (ART) potently suppresses HIV, to the point where it is difficult to detect in treated individuals. HIV does persist at low levels, however, and rebounds if ART is stopped. The state in which HIV persists is commonly thought to be invisible to immune responses, such as killer T-cells, which would otherwise eliminate infected cells. Efforts to cure HIV have therefore focused on developing strategies to expose these hidden cells to the immune system through ‘latency reversal’. We hypothesized that the concealment of the virus from T-cells in these individuals may not be absolute, and that a particular protein called ‘Nef’ may leave HIV partially exposed. We reasoned that, if this were true, we would observe an association between the strength of the T-cell response to Nef and the frequencies of HIV-infected cells. We tested this in population of 96 individuals on long-term ART. We observed a direct correlation between these two parameters, suggesting that Nef-specific T-cells continue to detect infected cells, but do not efficiently eliminate these exposed target cells. Our results suggest that boosting the killing ability of Nef-specific T-cells may reduce viral reservoirs, and thus contribute to achieving viral eradication or remission.

## Introduction

Antiretroviral therapy (ART) durably suppresses HIV replication, but does not lead to viral clearance. At least two mechanisms contribute to viral persistence. First, HIV establishes latent reservoirs in long-lived resting CD4^+^ T-cells, and potentially other cell types [1–3]. A paucity of proviral gene expression in these cells allows for their evasion of efficient recognition and clearance by the immune system [4]. This reservoir can be reactivated *ex vivo* by T-cell receptor (TCR) stimulation, mitogens, and potentially other latency reversing agents (LRAs) to produce infectious virus [5]. Second, viral expression persists in the B-cell follicles of lymph nodes, and potentially other anatomical sites, which are poorly accessible to cytotoxic T-lymphocytes (CTLs) [6–9]. A common assumption, consistent with these mechanisms of persistence, is that the infected cell population in individuals on long-term ART is invisible or inaccessible to CTLs. This has led to the “kick and kill” paradigm, which proposes to pair LRAs with CTLs, or other immune effectors, to reduce the number of HIV-infected cells [10–12]. More recently, considerable efforts have also shifted towards developing strategies to direct HIV-specific CTLs into lymph node follicles. It is postulated that combinations of strategies that address both proviral latency and anatomical sanctuaries may lead to reductions in viral reservoirs and long-term remission from viremia after cessation of ART.

Although latent reservoirs and compartmentalization are important mechanisms for HIV persistence, we questioned whether HIV-infected cells are completely invisible to the immune system in individuals on ART. As T-cells are able to detect even a single MHC-peptide complex on a cell surface [13], an exceptionally strict state of latency would need to be maintained for T-cell recognition of latently-infected cells to be completely absent. While both transcriptional initiation and elongation of proviral gene transcripts are severely impaired in resting CD4^+^ T-cells [14–16], both unspliced and multiply spliced HIV transcripts can be detected in these cells when assayed directly in peripheral blood mononuclear cells (PBMCs) of ART-treated individuals [16–19], suggesting the possibility of low-level antigen expression in the periphery. The exclusion of CD8^+^ T-cells from lymph node follicles is also not absolute, suggesting the likelihood of occasional interactions with cells actively expressing viral antigens in these compartments. The current study tests the hypothesis that ongoing interactions with HIV-infected cells continue to shape the HIV-specific T-cell response in individuals on long-term ART.

Upon initiation of ART, and once viremia is fully suppressed, HIV-specific CD8^+^ T-cell responses decay with a half-life of 39 weeks for at least 2 years [20–22]. This decay is very likely due to a reduction in, or perhaps an abrogation of HIV antigen expression. We reasoned that if low-level and/or episodic HIV antigen expression continued in individuals on long-term ART, then T-cell responses would be preferentially maintained in individuals with higher frequencies of persistently-HIV-infected cells. Of note, there have been recent observations that a subset of defective HIV proviruses, which make up the large majority of proviruses in ART-treated individuals, are capable of producing RNA transcripts [23], expressing antigens and inducing T-cell recognition [24]. These findings support the rationale for testing associations between HIV-infected cell frequencies (cell-associated HIV DNA [CA-HIV DNA]) and their transcriptional activity (cell-associated HIV RNA [CA-RNA]) with HIV specific T-cell responses, as opposed to only with the intact-inducible reservoir as measured by quantitative viral outgrowth assay (QVOA). To assess whether the immune response is sensing the infected cell population in individuals on ART, we evaluated whether there are correlations between T-cell responses against specific viral proteins and measures of the infected cell population in participants on long-term ART enrolled in a longitudinal AIDS Clinical Trials Group cohort study (ACTG A5321).

**Fig 1:**
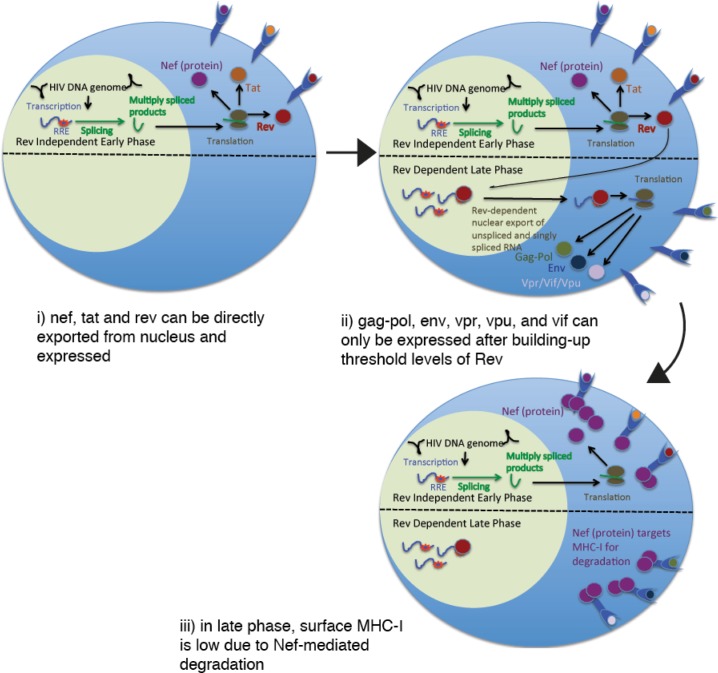
Rationale for hypothesizing preferential T-cell recognition of early versus late HIV gene products in ART-treated individuals.

Temporal control of the expression of different HIV gene products (Gag, Pol, Env, Nef, Tat, Rev, Vpr, Vpu, and Vif) from a single promoter (viral LTR) is achieved through an autoregulatory cascade (reviewed in [25]). Initially, cellular transcription factors drive the production of relatively low levels of HIV transcripts, of which only the multiply-spliced (ms) products Nef, Tat, and Rev have access to the cytoplasm, and thus to translational machinery [26–29]. Tat then initiates a positive feedback loop by facilitating the elongation of nascent HIV transcripts [30]. The switch from the expression of the “early” ms gene products Nef, Tat, and Rev, to the late unspliced (us) and single spliced (ss) gene products Gag-Pol, Env, Vif, Vpu, and Vpr is dependent upon the buildup of a threshold level of Rev protein, which acts to chaperone us and ss RNAs from the nucleus to the cytoplasm where they can be translated [27,28,31,32]. Based on the progressive nature of this expression cascade, we postulated that the early gene products Nef, Tat, and Rev would be more likely to be expressed by transiently or partially re-activated CD4^+^ T-cells as compared to the late gene products Gag, Pol, Env, Vif, Vpu, and Vpr. Additionally, since Nef is known to progressively downregulate MHC-I and MHC-II [33,34], an infected cell that is sufficiently reactivated to drive expression of late gene products will have lost a measure of antigenicity to T-cells by the time that this expression occurs. Expression of early gene products, in contrast, is initiated in the context of intact MHC expression, providing a transient window of relatively unimpaired antigen presentation. Based on the above lines of reasoning, our *a priori* hypothesis for the current study was that, in individuals on long-term ART, the magnitudes of T-cell responses to Nef, Tat, and Rev would correlate with frequencies of HIV-infected cells, while those to Gag, Pol, Env, Vif, Vpr, and Vpu would not. A visual summary of this hypothesis is presented in **Fig 1**. Interactions between T-cells and HIV-infected cells could result in opposing pressures on the directionality of any correlation: i) stimulation of T-cells by antigen could drive their maintenance and/or expansion, driving a direct correlation ii) HIV-infected cells could be eliminated by T-cells, driving an inverse correlation. Thus, overall, a direct correlation would suggest that interactions between HIV-specific T-cells and infected cells were dominated by expansion/maintenance of ineffective T-cell responses, whereas an inverse correlation would suggest the efficient clearance of infected cells. Appay *et al*, previously observed relatively strong Nef-specific T-cell responses in 3 of 4 individuals whom had been on an early ART regimen for 1–2 years and connected this with high levels of persistent *nef* RNA in the cohort as a whole–providing initial support for the hypothesis presented here and adding to the rationale for the current in-depth study.

## Results

### Nef-Specific T-cells recognize latently-infected and partially reactivated cells

**Fig 2:**
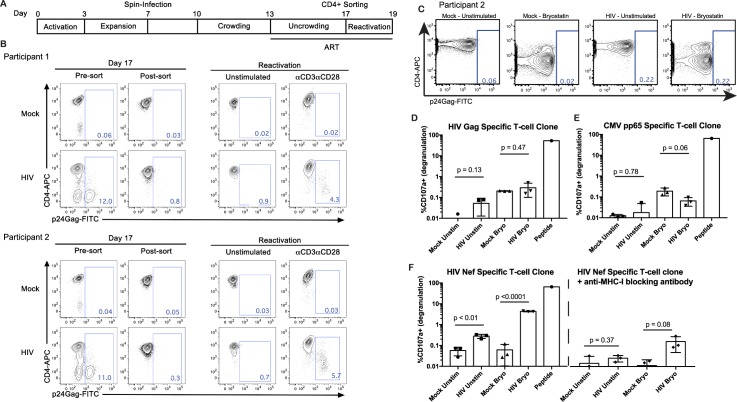
Comparing recognition of latently-infected and partially reactivated HIV-infected cells by Nef- and Gag-specific CD8^+^ T-cells. **A.** Timeline for generation of primary cell latency model. **B**. Characterization of latently-infected cells from two ART-treated participants showing a lack of Gag expressing cells post-sorting on Day 17, and reactivation of Gag expression from these populations following 48 hours of stimulation with anti-CD3/anti-CD28. **D**. Latently-infected cells from Day 17 (post-sort) were used for CD8^+^ T-cell recognition assays. Flow cytometry data from 10 hours of stimulation with bryostatin, or unstimulated controls, show a lack of detectable induction of Gag expression at this early time point. CD4 downregulation is observed as a result of bryostatin stimulation. Note that these are the same cells that show Gag expression following 48 hours of stimulation in **B**. **D—F.** Cells from this 10 hour stimulation time point were co-cultured with autologous HIV-Gag-specific (**D**), CMV-pp65-specific (neg control, **E**), or HIV-Nef-specific (**F**) CD8^+^ T-cell clones. Recognition of target cells by CD8^+^ T-cell clones was measured by degranulation (CD107a exposure). The results show recognition of latently-infected and partially reactivated cells by Nef-specific CD8^+^ T-cell clones, contemporaneous with a lack of detectable Gag expression and lack of recognition by Gag-specific CD8^+^ T-cells. Mean ± SD values are shown and p values were calculated by Student’s T test.

We first tested the plausibility of our hypothesis using a previously described primary cell latency model [35,36]. To generate this model, naïve CD4^+^ T-cells from two ARV-treated participants were stimulated for 3 days with anti-CD3/CD28 in the presence of TGF-β and blocking antibodies against IL-4 and IL-12 to induce maturation into non-polarized (NP) memory CD4^+^ T-cells. Cells were allowed to proliferate for 4 days, infected with replication competent HIV for 6 days, and then allowed to return to resting in the presence of ARVs for 4 days. Cells with persistent HIV expression were then depleted based on their CD4 surface expression, leaving purified populations of latently-infected cells mixed with uninfected cells (**Fig 2A**). Characterization of cells from both participants are shown in **Fig 2B**. Following 48 hours of anti-CD3/anti-CD28 stimulation in the presence of ARVs, 4.3% and 5.7% of cells were induced to express HIV-Gag, confirming the presence of previously latent populations of infected cells.

We isolated an HIV Nef-specific CD8^+^ T-cell clone (RMRRAEPAA epitope) from ART-treated Participant 1, as well as an HIV Gag-specific CD8^+^ T-cell clone (NTMLNTVGGH epitope) and a CMV pp65-specific CTL clone (epitope unknown) from Participant 2. These clones were used as sensors to detect the presentation of corresponding antigens in autologous latency model CD4^+^ T-cells, with the CMV-specific clone serving as a negative control. This is highly analogous to the method that we have used previously to assess CD8^+^ T-cell recognition of cells containing defective HIV proviruses [24], and to identify LRAs that induce HIV antigen expression [4]. Mock-infected or HIV-NL4-3-infected latency model CD4^+^ T-cells were either pulsed with 10 nM bryostatin for 2 hours or left as unstimulated controls. After an additional 8 hours, portions of these target cells were analyzed by flow cytometry, and the remaining cells were co-cultured with autologous CD8^+^ T-cell clones in the presence of Brefeldin A and Monensin. As Brefeldin A is a potent inhibitor of MHC-I antigen processing [37], antigen presentation profiles were arrested at this time point. We observed potent downregulation of CD4 by bryostatin stimulation, but this was not associated with the induction of HIV Gag expression at this early time point (**Fig 2C**) [note that these are the same target cells that exhibited 5.7% Gag^+^ cells after 48 hours of stimulation with anti-CD3/CD28 (**Fig 2B**)]. Corresponding with this, we observed a lack of recognition of HIV-infected cells in either the unstimulated or bryostatin treated conditions by the HIV-Gag specific CD8^+^ T-cell clone (**Fig 2D**). As expected, the negative control CMV-specific CD8^+^ T-cell clone also did not respond to HIV-infected cells (**Fig 2E**). Both clones potently degranulated in response to their cognate peptides, attesting to their specificities and functionalities (**Fig 2E**). In contrast, the HIV Nef-specific CD8^+^ T-cell clone exhibited detectable degranulation in response to unstimulated latently-infected cells, and potent degranulation in response to bryostatin stimulated infected cells (**Fig 2F**). These responses were largely abrogated by pre-treatment of target cells with a blocking anti-MHC-I antibody, further supporting the specificity of the assay **(Fig 2F**). Thus, in this primary cell model, Nef-specific CD8^+^ T-cells exhibited detectable recognition of antigen expression in latently-infected cells, and robust recognition after partial latency reversal, despite a lack of detectable Gag by either antibody staining or by a Gag-specific CD8^+^ T-cell clone. These results support a relative advantage of Nef-specific CD8^+^ T-cells in recognizing partial or early latency reversal (as in the first stage depicted in **Fig 1**). Note that we have previously demonstrated recognition of infected cells by Gag-specific CD8^+^ T-cell clones after longer periods of reactivation [4].

### IFN-γ responses to HIV Nef/Tat/Rev uniquely correlate with HIV DNA

**Fig 3:**
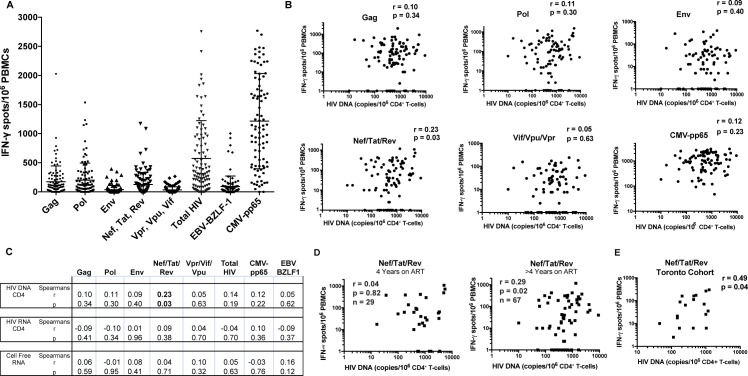
Magnitudes of T-cell responses to peptide pools, and correlations with virologic parameters. **A.** Peptide pools were tested in duplicate. CMV pp65 and EBV BZLF1 peptides are included as controls as responses to these other viruses are unlikely to be related to HIV reservoirs. Each data point represents the mean number of spots per 10^6^ PBMCs following background subtraction. Vertical lines and error bars represent mean and standard deviation for each peptide pool. **B**. Correlations between IFN-γ -producing HIV-specific T-cell responses (as displayed in **A**) and frequencies of HIV-infected cells as measured by cell associated HIV DNA, with each dot representing a single individual. Spearman’s r and p values are given for each peptide pool. **C**. Tests for correlations between T-cell responses and additional virologic parameters (cell-associated and cell-free HIV RNA). **D**. The ELISPOT data set depicted in **B** were split based on duration of ART (4 years, or >4 years). The results are consistent with the correlation between Nef/Tat/Rev-specific T-cell responses and HIV DNA emerging after years of ART. **E.** Correlations between IFN-γ -producing HIV-specific T-cell responses and frequencies of HIV-infected cells as measured by cell associated HIV DNA in the Toronto cohort.

To comprehensively assess HIV proteome-wide T-cell responses in the ACTG A5321 cohort (see **Table 1** for clinical data), we performed IFN-γ ELISPOT assays with overlapping peptide pools spanning: i) Gag ii) Env iii) Pol iv) Nef/ Tat/Rev v) Vif/Vpr/Vpu vi) CMV-pp65 (control) vii) EBV BZLF-1 (control). HIV-specific T-cell responses were primarily directed against Gag, Pol, and Nef/Tat/Rev with mean ± SD values of 171 ± 271, 295 ± 282, and 124 ± 205 SFU/10^6^ cells, respectively (**Fig 3A**). We observed a modest but significant correlation between the magnitude of the Nef/Tat/Rev-specific T-cell response and cell-associated HIV DNA (Spearman r = 0.23, p = 0.03) (**Fig 3B and 3C**). No correlations were observed between T-cell responses to the other HIV gene products and HIV DNA. Nef/Tat/Rev-specific T-cell responses did not significantly correlate with either CA-RNA or cell-free virion-associated RNA at the time of sampling (**Fig 3C**), or with pre-ART plasma HIV RNA (Spearman r = -0.15, p = 0.14) or CD4 counts (Spearman r = -0.03, p = 0.77). This correlation is consistent with our hypothesis that the early HIV gene products Nef, Tat, and Rev may be preferentially expressed in individuals on long-term ART. This hypothesis also predicts a strengthening of the correlation with longer durations of ART. To test this, we divided the population into individuals whom had been on ART for 4 years (n = 29) and those who had been on ART for > 4 years (5–15 years, n = 67). We observed a lack of correlation between Nef/Tat/Rev-specific T-cell responses in the former group (Spearman r = 0.04, p = 0.82, **Fig 3D**) and a strengthening of the correlation relative to the whole population in the latter group (Spearman r = 0.29, p = 0.02, **Fig 3D**). However, this difference in correlations was not statistically significant (p = 0.51, regression-based interaction test on ranks). Thus, our data are consistent with, but do not reliably confirm, that the relationship between Nef/Tat-Rev-specific T-cell responses emerges with longer durations of ART. The correlation between Nef/Tat/Rev-specific T-cells and HIV DNA was corroborated in a separate cohort of 18 ART-treated individuals from Toronto (Spearman r = 0.49, p = 0.04, **Fig 3E**), further supporting our primary hypothesis.

**Table 1:** Clinical characteristics of study participants.

	ACTG A5321 Cohort (n = 96)	Toronto cohort (n = 18)
Age at sample time point, median (range), years	43 (23–74)	47 (30–61)
Sex—female	26%	0%
Pre-therapy plasma HIV-1 RNA, median (range), log10 copies/ml	40,847 (190–979,159)	10,610 (<40–911,164)
Pre-therapy CD4+ T-cell count, median (range), cells/mm3	288 (0–734)	330 (52–460)
Years on therapy at sample time point, median (range), years	7 (4–15)	6 (2–25)
CD4 count at sample time point, median (range), cells/mm3	705 (149–1413)	650 (300–1000)

As an aside, no significant differences were observed between individuals on PI versus non-PI regimens with respect to the magnitudes of T-cell responses to any HIV gene products in this ACTG cohort (unpaired t tests: Gag, p = 0.87; Pol, p = 0.14; Nef/Tat/Rev p = 0.77; Env, p = 0.34; Vif/Vpr/Vpu, p = 0.93).

### Magnitude and breadth of Nef-specific T-cell responses correlate with HIV DNA

**Fig 4:**
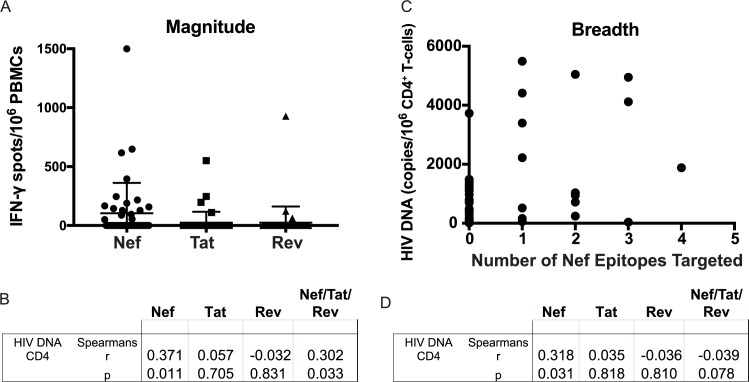
Magnitudes and breadths of Nef/Tat/Rev-specific T-cell responses, and correlations with virologic parameters. T-cell responses were measured by IFN-γ ELISPOT using a peptide matrix pool strategy (see Methods). **A.** Total magnitudes of responses to each gene product, calculated as sums of responses to individual matrix pools following background subtraction. Vertical lines and error bars represent mean and standard deviation for each gene product. **B**. Correlations between T-cell responses (as in **A**) and frequencies of HIV-infected cells as measured by cell associated HIV DNA. **C.** The total numbers of Nef epitopes recognized by each individual are shown plotted against frequencies of HIV-infected cells. **D.** Tests for correlations between breadths of T-cell responses (as in **C**) and frequencies of HIV-infected cells.

Using a matrix strategy with 19 peptide pools (covering 49 Nef peptides, 23 Tat peptides, and 27 Rev peptides), we next comprehensively mapped the peptides targeted by Nef, Tat, and Rev-specific T-cell responses in a subset of 50 samples, randomly selected from the 96 that were tested with peptide pools. Total magnitudes of responses to Nef, Tat, and Rev were determined by summing the magnitudes of individual epitope responses. Where responses mapped to two adjacent overlapping peptides (ex. Nef 14 & 15) this was considered as a single response (likely to an epitope shared by both 15mers) and only the stronger of the two was included in the summed response. While avoiding overestimation of responses, this conservative evaluation of data can potentially underestimate the total breadth and magnitude of responses, since more than one epitope can be contained within one overlapping peptide. Responses were predominately directed against Nef, with Tat- and Rev-specific responses infrequently detected (**Fig 4A**). The summed response to Nef/Tat/Rev correlated significantly with frequencies of infected cells as measured by HIV DNA (Spearman r = 0.30, p = 0.03) (**Fig 4B**), confirming the association that we initially observed with the Nef/Tat/Rev peptide pool. In breaking these down by gene product, we observed that this association was driven by T-cell responses to Nef (Spearman r = 0.37, Bonferroni-corrected p = 0.03), with no correlations observed for Tat (Spearman r = 0.06, p = 0.70) or Rev (Spearman r = -0.03, p = 0.83) (**Fig 4B**). As we did not establish an *a priori* hypothesis as to which of these three gene products would drive the Nef/Tat/Rev correlation it is appropriate to apply a Bonferroni correction to the unadjusted p value of 0.01, yielding a p value of 0.03 for the correlation with Nef-specific responses. Within Nef, the number of individual peptides targeted in a given individual ranged from 0 to 4. The breadth of this response also correlated directly with HIV DNA (Spearman r = 0.32, p = 0.03) (**Fig 4C and 4D)**. Thus, these data confirm a relationship between the frequencies of HIV DNA-harboring cells and Nef-specific T-cell responses in individuals on long-term ART.

### Polyfunctional profiling of HIV-specific CD8^+^ T-cell responses

**Fig 5:**
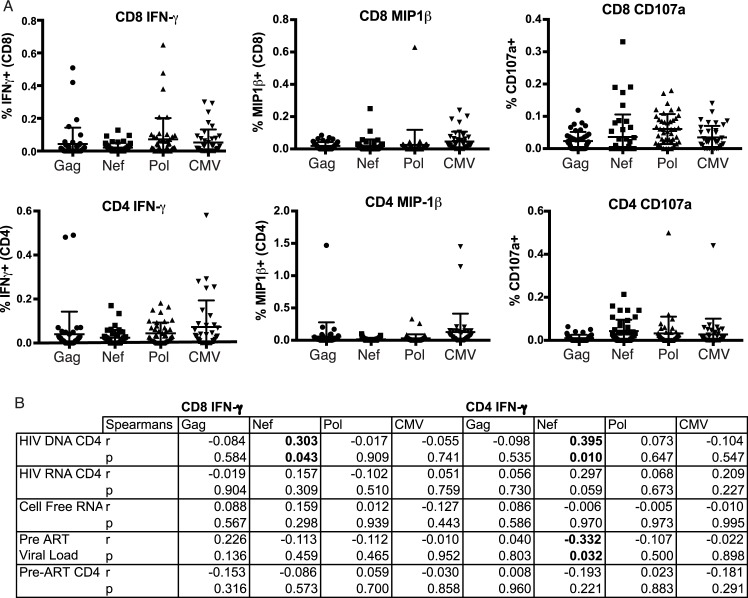
Polyfunctionality of HIV-specific T-cell responses, and tests for correlations with virologic parameters. **A.** Shown are frequencies of CD8^+^ and CD4^+^ T-cells that responded with the indicated effector functions following stimulation with HIV and CMV peptide pools. Each dot represents the background-subtracted mean response from a given individual (tested in duplicate). Vertical lines and error bars represent mean and standard deviation for each gene product. **B**. Tests for correlations between responses depicted in **A** and virologic parameters. Significant associations (p < 0.05) are indicated in bold.

Next, based on sample availability, we utilized intracellular cytokine staining flow cytometry to further assess the functionality of T-cell responses in 45 of the 50 samples that had undergone ELISPOT mapping. CD8^+^ and CD4^+^ T-cell responses that produced IFN-γ and degranulated (CD107a) were detected in response to HIV Gag, Pol, and Nef peptides. Lower magnitudes of MIP-1β-producing responses were detected, and only in a subset of individuals (**Fig 5A**). We observed significant direct correlations between frequencies of IFN-γ-producing Nef-specific T-cells in both the CD8^+^ and CD4^+^ compartments and HIV DNA (CD8: Spearman r = 0.30, p = 0.04; CD4: Spearman r = 0.40, p = 0.01), further corroborating our ELISPOT results. Also in accordance with the ELISPOT data, no other significant correlations were observed between the virological and immunological parameters measured in these experiments. However, there was a trend towards a direct correlation between cell-associated HIV RNA and CD4^+^ T-cells that produced IFN-γ in response to Nef (**Fig 5B**). The frequencies of IFN-γ-producing Nef-specific CD4^+^ T-cells, measured after long-term ART, did also correlate inversely with pre-ART HIV viral loads (p = 0.03, r = -0.33; **Fig 5B**). This latter observation is in agreement with an earlier study that reported an inverse correlation between IFN-γ-secreting Nef-specific CD8^+^ T-cell responses and HIV viral load in untreated infection [38].

## Discussion

Effective ART is commonly thought to sever the interactions between HIV and virus-specific immune responses, due to the elimination of antigen expression. The fundamental premise of “kick and kill” eradication strategies is therefore to re-engage the immune systems with viral reservoirs through the application of pharmacologic latency reversal, facilitating immune-mediated clearance. Few studies, however, have tested the possibility that some level of interaction may be ongoing due to low-level or episodic antigen expression. Recently, Lee *et al* reported a correlation between levels of antibody responses against the Gag matrix protein and integrated HIV DNA in resting CD4^+^ T-cells, and thus suggested that HIV-specific antibody responses could reflect the size of the HIV reservoir in treated individuals (antibodies to Nef were not tested) [39]. Similarly, Keating *et al*, have reported that, in a cohort of ART-treated individuals, HIV antibody levels correlated directly with frequencies of HIV-infected cells as measured by HIV DNA [40]. In these studies, as in ours, the immune system was used as a biosensor to indirectly assess viral antigen expression that could be: i) low level ii) anatomically localized iii) transient and sporadic over years of treatment, and thus difficult to measure directly. We also suggest that the latter two factors contribute to the observation that, both in these antibody studies and in the current study, immune responses were found to correlate with levels of HIV DNA, rather than RNA in peripheral blood CD4^+^ T-cells. We propose that: **i**) the total frequencies of HIV-infected cells (HIV DNA) may be a more reliable reflection of the cumulative residual antigen expression that maintains T-cell responses over years as compared with a snapshot of cell-associated HIV RNA levels, which may fluctuate or be comprised of hypermutant or otherwise defective message; **ii**) levels of cell-associated HIV RNA in peripheral blood may be a poorer reflection of RNA/antigen expression in tissue environments than total frequencies of infected cells, again due to fluctuations in expression of the former. In support of this latter point, the study by Lee *et al*, observed correlations between antibody responses and HIV RNA in cells from the gut-associated lymphoid tissue that were absent in comparison to peripheral blood HIV RNA; **iii**) a proportion of cell-associated HIV RNA is sequestered in the nucleus and thus not accessible to translational machinery [41]. We also note that evidence from an SIV-infected macaque model supports that CD8^+^ T-cells may suppress viral transcription without eliminating infected cells. Such an effect would act in opposition to a direct correlation with RNA, without affecting the relationship with DNA [42].

We acknowledge that the correlation that we observed between frequencies of HIV-infected cells and of IFN-γ-producing Nef-specific T-cell responses is modest (r = 0.37, p = 0.01). We had anticipated that any association, if present, would be difficult to detect for at least three reasons. First, as these individuals initiated treatment in chronic infection, a substantial proportion of their T-cell responses will be directed against epitopes that have acquired escape mutations [43,44], and thus will decay following suppression of viral replication regardless of any persistent antigen expression. Second, while we and others have recently highlighted the ability of a subset of defective proviruses to produce HIV transcripts and express antigens [23], and we have demonstrated that this can result in recognition by CD8^+^ T-cells, a large proportion of defective proviruses have no potential antigen expression (ex. large internal deletions spanning targeted epitopes) [45]. Thus, total HIV DNA is expected to be a relatively poor measure of antigen-expression potential. Third, we anticipate that many of the interactions between T-cells and antigen-expressing cells are likely to occur in lymphoid tissue rather than in blood. Neither the frequencies of HIV-infected target cells nor HIV-specific T-cells in the blood may be fully representative of the diverse tissues where interactions could take place. Our expectation of a weak correlation led us to study a relatively large number of individuals (n = 96). We also pre-specified our *a priori* hypothesis that only T-cell responses to Nef/Tat/Rev would correlate with frequencies of infected cells (the relevant section of ACTG New Works Concept Sheet is available upon request to the corresponding author). Though modest, the observed correlation was robust and was observed using two different ELISPOT strategies, as well as with intracellular cytokine staining on a sub-population of the ACTG cohort. It was also confirmed by ELISPOT using samples from in an independent cohort from Toronto.

By providing evidence that Nef-specific CD8^+^ T-cells continue to interact with HIV-infected cells in individuals on long-term ART, our study raises the provocative possibility that the immune system could be enlisted to drive some measure of reservoir reduction even in the absence of pharmacological latency-reversal. Our results suggest that T-cells targeting Nef may be uniquely able to recognize persistent antigen expression, possibly due to a lower threshold for expression than Rev-dependent gene products such as Gag, Pol, and Env. Nef-mediated MHC-I / MHC-II downregulation, which serves to diminish T-cell recognition of infected cells, may also differentially impact T-cell responses to early versus late gene products. While responses to both may ultimately be negatively impacted, the expression of late gene products occurs only after MHC-I / MHC-II downregulation, whereas early gene product expression initiates in the context of intact MHC-I / MHC-II expression and thus has a window of opportunity for relatively unimpaired presentation following reactivation (model presented in **Fig 1**). Critically, however, we observed a direct rather than inverse relationship between Nef-specific T-cell responses and frequencies of infected cells. Our results therefore suggest that the recognition of infected cells does not lead to effective clearance, and thus that simply boosting the magnitudes of Nef-specific T-cell responses is unlikely to be an effective strategy for reducing viral reservoirs. Rather, immunotherapeutic strategies that aim to boost the ‘quality’ and function of Nef-specific CD8^+^ T-cell responses should be explored. As one example, we have previously demonstrated that treatment of *ex vivo* CD8^+^ T-cells from ART-treated HIV-infected individuals with an IL-15 superagonist enhances their abilities to kill autologous HIV-infected CD4^+^ T-cells [4]. This is likely due, at least in part, to the ability of IL-15 to induce the upregulation of the cytolytic effector molecule perforin, and to enhance T-cell receptor mediated degranulation [46]. While potent combination strategies that incorporate effective latency-reversing agents will very likely be needed to achieve eradication or remission, our study suggests the potential to drive some level of reservoir reduction by enhancing the “kill” alone. Our results lead us to hypothesize that this may be achievable by the focused boosting or elicitation of T-cell responses against early, Rev-independent gene products–Nef in particular–in conjugation with immunotherapy to enhance the cytotoxic capacity of these cells.

## Materials & methods

### Study population

We evaluated participants from two separate populations. The data in **Fig 3E** relate to a population recruited through the Maple Leaf Medical clinic in Toronto, Canada. Participants in this Toronto cohort were on ART regimens for a minimum of 2 years prior leukapheresis with no reported ART interruptions. Peripheral blood mononuclear cells and plasma were obtained from 18 male participants whom have been suppressed on ART for a median of 6 years (range 2–25 years). Median age was 47 years (range 30–61) with median pre-treatment HIV viral loads of 10,610 copies/mL (range <40–911,164 copies/mL) and CD4 counts during draw of 650 cells/mm^3^ (range 300–1000 cells/mm^3^). Median CD4 counts pre-therapy was 330 cells/mm^3^ (range 52–460 cells/mm^3^). 6 participants were on ARV regimens containing protease inhibitor up to time of leukapheresis while 12 were on non-PI regimens.

All other data pertain to a population of participants with chronic HIV infection who initiated ART in AIDS Clinical Trials Group (ACTG) trials for treatment-naïve individuals and had subsequent follow-up while continuing to receive ART (ACTG studies A5001 and A5321). Participants in the current study had plasma HIV RNA levels < 50 copies/mL by commercial assays starting at week 48 of ART and at all subsequent time points and no reported ART interruptions. Peripheral blood mononuclear cells and plasma were obtained from 96 of these individuals (25 women and 71 men) whom had been suppressed on ART for a median of 7 years (range 4–15 years). Median age was 43 years (range 23–74 years) with median pre-treatment HIV viral loads of 40,847 copies/ml (range 190–979,159 copies/ml), and pre-treatment CD4 counts of median 288 cells/mm^3^ (range 0–734 cells/mm^3^). 35 participants were on ARV regimens containing protease inhibitors (PI) while 61 were on non-PI regimens. For individuals on non-PI regimens, 35 were treated with efavirenz, 2 with nevirapine, 6 with rilpivirine, and 18 with raltegravir.

### Ethics statement

The study was approved by ethics committees at each participating ACTG site, at the University of Toronto, and at the authors’ institutions. Written informed consent was obtained from each participant.

### Generation and maintenance of CD8^+^ T-cell clones

CD8^+^ T-cell responses in study participants were mapped by IFN-γ ELISPOT using 270 previously defined HIV optimal epitopes restricted by common HLA alleles. For each response, PBMC were plated at 1x10^7^ cells/well in a 24-well plate and stimulated with 10 μg/ml of peptide for 3 hours. T-cells that had produced IFN-γ in response to this stimulation were enriched using the IFN-γ secretion detection and enrichment kit (Miltenyi Bioetc) following the manufacturer’s instructions. These cells were plated at a series of dilutions in 96-well plates with feeder medium (RPMI 1640 supplemented with 10% FBS and PenStrep [R-10] with 1x10^6^ cells/ml 5,000 rad irradiated PBMC + 50 U/ml IL-2 + 0.1 μg/ml each of anti-CD3 (OKT3, ebioscience), anti-CD28 (CD28.2, ebioscience). One month later, colonies were selected from the lowest dilution plate with positive wells (<1/5 of wells positive) and screened for responsiveness to peptide by IFN-γ ELISPOT. Positive clones were expanded bi-weekly with feeder medium.

### Generation of primary cell latency model

Peripheral blood mononuclear cells were obtained from leukapheresis of ART-treated HIV-infected individuals. Cultured T_CM_ and latently infected cultured T_CM_ were generated as previously described [36,47,48].

### CD8^+^ T-Cell clone recognition assays

Latency model cells were obtained at day 17 of the experimental timeline shown in **Fig 2A** (immediately after depletion of productively-infected cells). These were pulsed for 2 hours with 10 nM bryostatin-1 (Sigma) in R-10, or maintained as unstimulated controls under matched conditions. Stimulated and unstimulated cells were then washed 4x with 14 ml R-10 (to remove bryostatin) and placed in R-10 for an additional 8 hours. HIV-specific CD8^+^ T-cells clones autologous to these targets were generated as described above. These were washed 3x with 14 ml R-10 and then co-cultured with target cells at a ratio of ~1:1 in R10 + 50 U/ml IL-2 + 1 μg/ml Brefeldin A + 1 μg/ml Monensin + 1/100 dilution of anti-CD107a-PE. Note that Brefeldin A is potent inhibitor of MHC-I antigen processing [37], thus antigen presentation profiles of target cells were arrested at this time point. Co-cultures were incubated at 37°C, 5% CO_2_ for 8 hours. Cells were then surface stained with amine-aqua viability dye (Life Technologies), anti-CD4 Pacific Blue (Biolegend), anti-CD8 AlexaFluor 700 (Biolegend), and anti-CD3 BV785 (Biolegend). Cells were then fixed in 2% paraformaldehyde and analyzed on a Fortessa flow cytometer (BD). Recognition of target cells by CD8+ T-cells was assessed by measuring CD107a (degranulation) within the viable (amine-aqua^-^) CD3^+^CD8^+^ population.

### Peptide pools

The following sets of consensus HIV Clade B 15 amino acid peptides overlapping by 11 amino acids were obtained from the NIH AIDS Research and Reference Reagent Program: Gag (cat # 8117), Pol (cat # 6208), Nef (cat # 5189), consensus B Tat (cat # 5138), Vif (cat # 6446), Rev (cat # 6445), Vpr (cat # 6447), Vpu (Cat # 6444), Env (Cat # 9480). All peptides were dissolved at 20 mg/ml in 100% DMSO (Hybri-Max™, Sigma-Aldrich). For each of Gag, Pol, and Env–peptides were pooled and concentrations were adjusted to 200 μg/ml/peptide in PBS. Nef, Tat, and Rev peptides as well as Vpr, Vpu, and Vif peptides were pooled together and adjusted to 200 μg/ml/peptide in PBS. A CMV-pp65 PepMix™ peptide pool and an EBV BZLF-1 PepMix™ peptide pool (JPT Peptide Technologies) were dissolved separately in DMSO, and adjusted to 20 μg/ml/peptide in PBS.

### IFN-γ ELISPOT

Multiscreen IP 96-well plates (Millipore) were coated with 100 μl/well of PBS + 0.5 μg/ml primary anti-IFN-γ antibody (clone 1-D1K, Mabtech) overnight. Plates were washed 6x with sterile 1% fetal bovine serum (FBS) (Gibco) in PBS. PBMCs were thawed, resuspended in RPMI 10% FBS (Gibco) (‘R-10’) and added to plates at 200,000 cells/well in 95 μl/well of R-10 medium. HIV peptide pools (200 μg/ml/peptide stock in 5% DMSO) were added at 5 μl/well to give final concentrations of 10 μg/ml/peptide in <0.5% DMSO. CMV-pp65 and EBV-BZLF1 peptide pools were added at 10 μl/well to give final concentrations of 1 μg/ml/peptide in 0.25% DMSO. PHA was added at 2 μg/ml as a positive control. Plates were incubated at 37°C, 5% CO_2_ for 18 hours. Plates were washed 6x with PBS. Secondary antibody–clone 7-B6-1 (Mabetch) was diluted to 0.5 μg/ml in PBS and added at 100 μl/well for 1 hour. Plates were then washed with PBS and incubated with 0.5 μg/ml Streptavidin-ALP (Mabtech) diluted in PBS for 1 hour in the dark. Plates were then washed 6x with 200 μl/well PBS. Color development solution: 9.6 ml ddH20, 400 μl 25x AP Color Development Buffer (Biorad), 100 μl Color Reagent A (Biorad), 100 μl Color Reagent B (Biorad) was added to the plate at 100 μl/well for 15 minutes. After removal of the color development solution, 100ul of 0.5% Tween-20 in PBS was added to each well for 10 mins. Plates were then washed with water, dried overnight and counted. A positive response was considered as one which met both of the following two criteria: 1) >50 spot forming units (sfu)/million PBMC after background subtraction 2) >2x above background.

### Peptide pool matrix mapping

For the epitope mapping experiments shown in **Fig 4**, Nef, Tat, and Rev peptides were arranged into 20 pools of 9–10 peptides each, such that every peptide was uniquely present in two different pools, allowing for the identification of individual peptide responses as previously described [49]. ELISPOT assays were performed as above using these 20 pools. The total magnitudes of responses to Nef, Tat, and Rev were determined by summing the individual peptide responses indicated by matrix pools. Breadths of responses were defined as the numbers of unique epitopes indicated by the matrix mapping–where responses mapped to two adjacent overlapping peptides (ex. Nef 14 & 15) this was considered as a single response.

### Intracellular cytokine staining

PBMCs were thawed, resuspended in R-10 medium and added to 96-well plates at 500,000 cells/well in 200 μl/well of R-10 medium. Peptides were added at the same concentrations as in ELISPOT assays for 6 hour stimulations in the presence of 1 ug/ml each brefeldin A (GolgiPlug, BD Biosciences) and monensin (GolgiStop, BD Biosciences), and 2 μl/well of anti-CD107a-PerCP-Cy5.5 (clone H4A3, Biolegend). Cells were then washed in 1% FBS PBS and surface stained with fluorochrome-conjugated antibodies to CD3 Brilliant Violet 711 clone OKT3, CD4 Brilliant Violet 650 clone OKT4, CD8-Alexa Fluor 700 clone HIT8a, CD62L-PE-Cy5 clone DREG-56, CD45RA-Alexa Fluor 488 clone HI100, PD-1-Brilliant Violet 605 clone EH12.2H7 (all from Biolegend), Tim-3-PE (clone 344823, R&D Systems), as well as fixable aqua viability dye (Life Technologies). Cells were fixed and permeabilized using the BD Cytofix/Cytoperm system, following the manufacturer’s instructions. Permeabilized cells were then stained with anti-IFN-γ-APC (BD Pharmingen, clone B27) and anti-MIP-1-β-APC-H7 (BD Pharmingen, clone D21-1351). Cells were analyzed on a BD LSR Fortessa X20 flow cytometer with FACSDIVA software. Data were analyzed using FlowJo software, TreeStar.

### Virologic assays

*ACTG samples*: Cell-associated HIV DNA and cell-associated HIV RNA (CA-RNA) were measured by quantitative PCR (qPCR) in PBMC samples using methods that have been previously published [50]. PBMC samples from each participant were thawed and assayed in the same qPCR run. Plasma HIV RNA by single-copy assay (SCA) was measured using methods that have been previously published [51]. Primers and probes used for qPCR of HIV DNA, CA-RNA and plasma HIV RNA were identical [50]. HIV DNA and CA-RNA values per million CD4^+^ T-cells were calculated by dividing the total HIV DNA or CA-RNA copies/million PBMCs (normalized for CCR5 copies measured by qPCR as published [50], by the CD4^+^ T-cell percentage (x 0.01) reported from the same specimen date or from a CD4^+^ T-cell percentage imputed using linear interpolation from specimen dates before and after the HIV DNA or CA-RNA results.

*Toronto samples*: Digital droplet PCR was performed as previously described[52]. Genomic DNA was extracted using the Gentra Puregene kit (Gentra) following the manufacturer’s instructions. For each PCR reaction, 5 units of restriction enzyme BsaJI (NEB) was directly mixed with 300ng of DNA, ddPCR Supermix for Probes (Bio-Rad), and final concentrations of 900nM primers and 250nM probe. Primers/Probes were: RPP30 –fprimer GATTTGGACCTGCGAGCG, rprimer GCGGCTGTCTCCACAAGT, probe VIC-CTGAACTGAAGGCTCT-MGBNFQ; HIV-gag–fprimer TACTGACGCTCTCGCACC, rprimer TCTCGACGCAGGACTCG, probe FAM-CTCTCTCCTTCTAGCCTC-MGBNFQ. Droplets were prepared using the QX100 Droplet Generator (Bio-Rad) following the manufacturer’s instructions. Sealed plates were cycled using the following program: 95°C for 10 min; 40 cycles of 94°C for 30 s, 60°C for 1 min; and 98°C for 10 min, with 2°C/sec ramping speed to ensure even droplet heating. Reactions were analyzed using the QX100 Droplet Reader, and template molecules per μL of starting material were estimated using the Quantalife ddPCR software. Eight replicates were run for each sample. We consistently applied a pre-determined exclusion criteria to outliers that deviated from mean values by >2x the standard deviation.

### Statistics

Statistical analyses were performed using Prism Software (GraphPad). Descriptions of the tests used are given in Results and in Figure Legends.
